# Clinical Characteristics of Dengue Shock Syndrome in Vietnamese Children: A 10-Year Prospective Study in a Single Hospital

**DOI:** 10.1093/cid/cit594

**Published:** 2013-09-17

**Authors:** Phung Khanh Lam, Dong Thi Hoai Tam, Tran Vinh Diet, Cao Thi Tam, Nguyen Thi Hanh Tien, Nguyen Tan Thanh Kieu, Cameron Simmons, Jeremy Farrar, Nguyen Thi Ngoc Nga, Phan Tu Qui, Nguyen Minh Dung, Marcel Wolbers, Bridget Wills

**Affiliations:** 1 Oxford University Clinical Research Unit, Hospital for Tropical Diseases; 2 University of Medicine and Pharmacy of Ho Chi Minh City; 3 Hospital for Tropical Diseases, Ho Chi Minh City, Vietnam; 4 Centre for Tropical Medicine, Centre for Clinical Vaccinology and Tropical Medicine, University of Oxford, United Kingdom

**Keywords:** dengue shock syndrome, pediatrics, Vietnam, prospective descriptive study

## Abstract

***Background.*** Dengue shock syndrome (DSS) is a severe manifestation of dengue virus infection that particularly affects children and young adults. Despite its increasing global importance, there are no prospective studies describing the clinical characteristics, management, or outcomes of DSS.

***Methods.*** We describe the findings at onset of shock and the clinical evolution until discharge or death, from a comprehensive prospective dataset of 1719 Vietnamese children with laboratory-confirmed DSS managed on a single intensive care unit between 1999 and 2009.

***Results.*** The median age of patients was 10 years. Most cases had secondary immune responses, with only 6 clear primary infections, and all 4 dengue virus serotypes were represented during the 10-year study. Shock occurred commonly between days 4 and 6 of illness. Clinical signs and symptoms were generally consistent with empirical descriptions of DSS, although at presentation 153 (9%) were still febrile and almost one-third had no bleeding. Overall, 31 (2%) patients developed severe bleeding, primarily from the gastrointestinal tract, 26 of whom required blood transfusion. Only 8 patients died, although 123 of 1719 (7%) patients had unrecordable blood pressure at presentation and 417 of the remaining 1596 (26%) were hypotensive for age. The majority recovered well with standard crystalloid resuscitation or following a single colloid infusion. All cases were classified as severe dengue, while only 70% eventually fulfilled all 4 criteria for the 1997 World Health Organization classification of dengue hemorrhagic fever.

***Conclusions.*** With prompt intervention and assiduous clinical care by experienced staff, the outcome of this potentially fatal condition can be excellent.

## Abstract (precis)

We present the clinical features, management, and outcomes for 1719 Vietnamese children with dengue shock syndrome enrolled in a 10-year prospective study in a single center, to provide the first comprehensive description of this increasingly important disorder.

Dengue is the most important mosquito-borne viral disease affecting humans. Infection can be caused by any 1 of 4 dengue viruses (DENV-1 to DENV-4), transmitted by *Aedes* mosquitoes [1]. Currently about 100 million clinically apparent cases are estimated to occur each year [2], resulting in approximately 20 000 deaths [3].

Infection with any serotype can cause a broad range of disease manifestations, from inapparent infection to severe and fatal disease [4]. The most notable complication is an unexplained vasculopathy that manifests as a transient increase in vascular permeability resulting in leakage of plasma from the circulation. Substantial plasma losses may occur, leading to the potentially fatal dengue shock syndrome (DSS). Although adults do experience shock, vascular leakage is generally more severe in young children [5], and in endemic areas DSS is seen primarily in the pediatric population. Thrombocytopenia and coagulation derangements also occur, and a variety of bleeding manifestations ranging from minor skin petechiae to major mucosal bleeding may be seen. Neither vaccines nor specific therapies are currently available; careful clinical observation and judicious use of intravenous fluid therapy, in particular urgent shock resuscitation for DSS, are the foundations for successful management [6]. Many individuals with DSS respond to resuscitation with crystalloid solutions, but patients with profound or protracted shock often require additional support with colloid solutions and are at risk of developing respiratory compromise due to ongoing plasma leakage. Mortality rates for DSS vary from <1% to >10% depending on the severity of the cases reported, the level of monitoring available, and the experience of the attending healthcare personnel [7–9].

Despite the increasing burden of dengue globally, only a few small retrospective reports have described the clinical characteristics, management, or outcomes of DSS [8, 10, 11]. At the Hospital for Tropical Diseases (HTD) in Ho Chi Minh City, a prospective observational study aiming to enroll all children presenting with DSS was conducted from 1999 to 2009. Here we present data on >1700 cases collected during this 10-year period, providing the first comprehensive description of the clinical features of DSS in children.

## METHODS

### Study Design and Participants

At HTD, children aged <15 years with DSS are managed on the pediatric intensive care unit (PICU). In 1999, we commenced a prospective observational study in which children admitted with clinically diagnosed DSS—that is, a history consistent with dengue, with hemodynamic compromise (either narrowing of the pulse pressure or hypotension for age, with evidence of impaired perfusion) thought by the treating clinician to be due to vascular leakage and to require volume resuscitation—were eligible to participate. Patients transferred from other facilities for tertiary care after initial resuscitation were not eligible. A double-blind randomized controlled trial (RCT) that was conducted within the study period (1999–2004) comparing different fluid solutions for initial resuscitation has been reported previously [7]. Both studies were approved by the HTD ethical committee and the Oxford Tropical Research Ethics Committee.

### Study Procedures and Data Collection

Following written informed consent by a parent or guardian, trained study physicians enrolled participants immediately after diagnosis of shock. Demographic characteristics, clinical history, and examination findings were recorded on a structured case report form at enrollment and then daily until discharge or death, together with detailed information about all therapeutic interventions and supportive care required. Specimens for dengue diagnostics were obtained at enrollment and discharge. All survivors were asked to return for follow-up assessment 1 month later.

A core group of senior clinicians supervised patient care throughout the study period, following the management guidelines for pediatric DSS at HTD. The routine initial regimen consisted of 25 mL/kg Ringer's lactate solution over 2 hours, with colloid solutions (dextran or starch) reserved for children presenting with profound shock. However, during the RCT, patients were randomized to receive 1 of these 3 fluid solutions, at the same rate but blinded, for their initial resuscitation [7]. A standardized schedule of Ringer's lactate was then used for all patients, involving staged reductions at specific time intervals, aiming for maintenance fluid therapy after 8 hours. Patients who failed to stabilize within 2 hours, or who deteriorated during the mandatory 36- to 48-hour period of close observation, received 10–15 mL/kg infusions of rescue colloid plus inotropes, blood products, or other therapy at the discretion of the treating clinician.

Cardiovascular status was monitored at least hourly until stable for 24 hours, and subsequently every 4 hours. The capillary hematocrit was measured at baseline, approximately 2 and 6 hours after study entry, and then every 12 hours or in the event of cardiovascular deterioration. A complete blood count was performed once daily, whereas other laboratory tests (eg, liver/renal function) were checked on clinical grounds rather than according to a defined study schedule. Disease classification was performed using the World Health Organization (WHO) 1997 and 2009 criteria (Supplementary Appendix 1) [4, 12].

### Dengue Diagnostics

Dengue immunoglobulin M and immunoglobulin G (IgG) capture enzyme-linked immunosorbent assays were performed on paired enrollment and early convalescent specimens, together with reverse transcription polymerase chain reaction (RT-PCR) on the enrollment specimen [13–15]. Seroconversion and/or detection of DENV RNA in plasma defined laboratory-confirmed cases (Supplementary Appendix 2). A positive dengue-specific IgG on or before day 7 of illness defined a secondary infection, whereas 2 negative dengue-specific IgG results, at least 1 obtained after day 7, were required to define a primary infection.

### Statistical Analysis

Continuous and categorical variables were summarized as median and interquartile range (IQR), or frequency and percentage, respectively. All analyses were performed with the statistical software R, version 2.15.0 [16].

## RESULTS

From 1999 to 2009, a total of 1810 of 1847 children (98%) admitted to PICU with clinical DSS participated in the study. In 19 cases, both RT-PCR and paired serology were negative, whereas in 72 cases the results were inconclusive; in the remaining 1719 cases (95%) dengue was confirmed, with the infecting serotype identified in 1209 of 1647 cases (73%) for which RT-PCR was performed. Among the confirmed dengue patients, 503 (29%) participated in the RCT, with the remainder enrolled in the observational study. Almost all cases came from the local catchment area, with <5% of cases transferred from elsewhere; however, 2 patients were enrolled in error, having already received parenteral fluids for shock resuscitation prior to transfer.

### Characteristics at Presentation With Shock

Demographic information and selected clinical characteristics for all 1719 patients with confirmed dengue are described in Table 1. For most parameters, data were missing in <5% of cases. The median age was 10 years, varying by year of study from age 9 to 11 years. The median day of illness at shock was consistently 5 (IQR, 4–6) for each study year, although 62 patients (4%) overall presented on illness day 3.

**Table 1.** Baseline Characteristics of the Study Participants at Enrollment

Characteristic	No.	Median/Frequency	IQR/%
Demographic characteristics			
Age, y	1719	10	(7–12)
Male sex	1719	902	(52)
Referral status	1719		
Directly from home		720	(42)
From other wards in HTD		911	(53)
From other healthcare centers		65	(4)
Unknown		23	(1)
Clinical and basic laboratory features			
Day of illness	1719	5	(4–6)
Weight, kg	1719	27	(20–35)
Temperature ≥38°C	1718	153	(9)
Pulse rate per min, if measurable^a^	1393	120	(100–120)
Systolic blood pressure, mm Hg, if measurable^a^	1596	90	(80–100)
Pulse pressure, mm Hg, if measurable^a^	1576	20	(15–20)
Hemorrhage	1719		
None		493	(29)
Skin only		1153	(67)
Mucosal		73	(4)
Abdominal tenderness	1714	1238	(72)
Liver palpable	1696	1478	(87)
Hematocrit, %	1696	49	(46–52)
Platelet count, cells/µL	1695	41 000	(28 000–61 000)
Aspartate aminotransferase, IU/L	1030	125	(80–206)
DHF according to WHO 1997 criteria^b^	1642	939	(57)
Dengue diagnostic tests			
RT-PCR performed	1647		
DENV-1		675	(41)
DENV-2		367	(22)
DENV-3		48	(3)
DENV-4		110	(7)
Mixed		9	(1)
Negative		438	(27)
Immune status	1618		
Primary		6	(<1)
Secondary		1506	(93)
Unclassifiable		106	(6)

Continuous variables are summarized as median (interquartile range) and categorical variables as frequency (%).

Abbreviations: DENV, dengue virus; DHF, dengue hemorrhagic fever; HTD, Hospital for Tropical Diseases; IQR, interquartile range; RT-PCR, reverse transcription polymerase chain reaction; WHO, World Health Organization.

^a^ All patients were assessed for these parameters, but we only report values for patients in whom the parameter could be measured. Note that in some patients a systolic pressure could be detected but the pulse was too rapid to count.

^b^ We used data available at the time of presentation with shock only. Tourniquet tests were not routinely performed. For each patient, the baseline hematocrit level was defined using local population values taken from an unpublished dataset including >1000 healthy Vietnamese children (37% if aged ≤10 years, 38.5% if female aged >10 years, 40% if male aged >10 years).

Commonly reported symptoms included lethargy (1490/1719 [87%]), vomiting (1199/1713 [70%]), and abdominal pain (932/1709 [55%]). Most children were afebrile, but 153 of 1718 (9%) still had an axillary temperature of ≥38°C at onset of shock, without a clear relationship to the day of illness (*P* = .09, Wilcoxon rank-sum test). In 123 of 1719 (7%), no blood pressure was measureable, whereas 417 of 1596 (26%) of the remainder exhibited hypotension for age, and 1568 of 1596 (98%) had a pulse pressure of ≤20 mm Hg. Respiratory distress (3/1718 [<1%]) and cyanosis due to profound shock (10/1714 [<1%]) were extremely uncommon. The liver was palpable in 1478 of 1696 (87%) of cases, with abdominal tenderness in 1238 of 1714 (72%), whereas a palpable spleen was extremely uncommon (only 5 cases documented). Almost one-third (493/1719 [29%]) of the patients had no bleeding. Among cases with bleeding, this amounted to skin petechiae or minor bruising in the majority, with mucosal hemorrhage noted in only 73 cases.

### Progress in Hospital

Because many patients in the RCT were randomized to a colloid for initial resuscitation, information on management and complications after enrollment is presented for the observational study and RCT groups separately (Table 2). Apart from the greater colloid usage, there was little difference between the 2 study groups other than a slightly higher proportion of minor skin bleeding observed in the RCT group. Considering the observational study only, most children recovered well with standard crystalloid resuscitation, although 547 of 1211 (45%) patients also received colloid therapy, 244 (45%) of them within the first 2 hours. Most children (328 [60%]) in this group received only a single colloid bolus, but up to 7 colloid infusions were needed for severe cases, with a median volume of 19 mL/kg (IQR, 13–25 mL/kg) of colloid given throughout hospitalization, on a background of 114 mL/kg (IQR, 99–129 mL/kg) total parenteral fluid therapy. Considering the whole patient cohort, additional cardiovascular support with inotropic drugs was required in 75 of 1719 patients (4%), and 513 of 1717 (30%) patients developed signs of fluid overload (overt pleural effusion or ascites) following resuscitation. Among these patients, 313 of 513 (61%) were treated with diuretic therapy for 1–2 days after hemodynamic stabilization.

**Table 2.** Summary of Complications, Management, and Outcomes

	Observational Study (n = 1216)	RCT (n = 503)	All Patients (N = 1719)
New bleeding	77 (6)	81 (16)	158 (9)
None	1138 (94)	423 (84)	1561 (91)
Skin only	39 (3)	59 (12)	98 (6)
Mucosal	38 (3)	22 (4)	60 (3)
Severe bleeding	20 (2)	11 (2)	31 (2)
Transfusion	18 (1)	8 (2)	26 (2)
Inotropic support	55 (5)	20 (4)	75 (4)
Total parenteral fluid volume, mL/kg^a^	114 (99–129)	125 (110–143)	116 (102–133)
Received colloid^a^	547 (45)	418 (83)	965 (56)
Total colloid volume^b^, mL/kg	19 (13–25)	25 (24–25)	25 (15–29)
Clinical fluid overload^a,c^	340 (28)	173 (34)	513 (30)
DHF according to WHO 1997 criteria^a,d^	796 (66)	406 (81)	1202 (70)
Death	7 (<1)	1 (<1)	8 (<1)

Continuous variables are summarized as median (interquartile range) and categorical variables as frequency (%).

Abbreviations: DHF, dengue hemorrhagic fever; RCT, randomized controlled trial; WHO, World Health Organization.

^a^ Up to 14 missing values.

^b^ Only includes patients who received a colloid infusion.

^c^ Clinically detectable pleural effusion or ascites.

^d^ Using all available acute and convalescent information.

After admission, 158 of 1719 (9%) children developed at least 1 new bleeding manifestation, among them 98 cases with skin bleeding only and 60 cases with mucosal bleeding. Considering all 126 patients with overt mucosal bleeding, gastrointestinal bleeding occurred most frequently (n = 61), compared to epistaxis (n = 36), gum bleeding (n = 22), or unusual vaginal bleeding (n = 21). The bleeding was clinically severe in 31 cases, 26 requiring transfusion (18 during active resuscitation, and 8 during the recovery phase), 4 resulting in compensated anemia at discharge, and 1 case involving a critical organ (spinal cord hemorrhage, confirmed by magnetic resonance imaging). Although most severe bleeding primarily involved the gastrointestinal tract (n = 15), 7 children had isolated severe skin bleeding, mainly at sites of invasive procedures, and 4 of 7 required transfusion. Platelet concentrates were not available during the study, but children with severe coagulopathy and active bleeding received fresh frozen plasma or other blood products at the discretion of the treating clinician.

**Figure 1.**
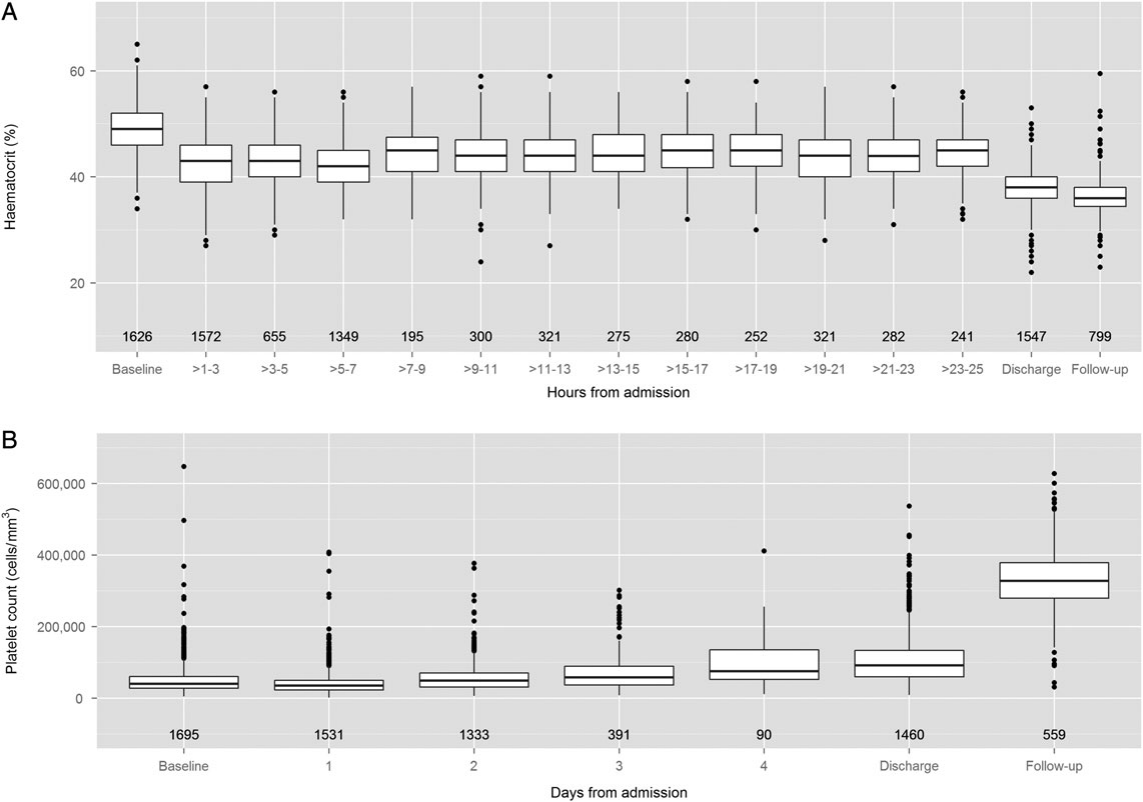
Box plots describing changes in hematocrit (*A*) and platelet count (*B*) during the evolution of the illness. Hematocrit data are presented for the 24 hours following admission, whereas platelet data are presented daily for the first 4 days, together with the discharge day and follow-up values for both parameters. The numbers displayed below each box plot represent the number of patients included within that time interval. If multiple values were recorded during any time interval, we chose the highest hematocrit and the lowest platelet count, respectively, for that patient. The hematocrit graph excludes data from the 73 patients with dengue shock syndrome with mucosal bleeding at presentation.

The evolution of hematocrit and platelet values is shown in Figure 1. The median maximum hematocrit was 50% (IQR, 47%–52%), documented at presentation in most cases (1484/1719 [86%]). Among patients with both enrollment and 1-month follow-up values, 755 of 832 (91%) had at least 20% hemoconcentration at enrollment. The hematocrit declined rapidly during the first 4 hours of resuscitation, later rising again in the majority of children. In contrast, the platelet nadir (median, 28 000 cells/µL [IQR, 19 000–40 000 cells/µL]) occurred most frequently 1 day after onset of shock (720/1718 [42%]). Although a transient drop in platelet count was seen in all cases, in 25 of 1718 cases the nadir remained >100 000 cells/µL. Coagulation profiles were performed infrequently and are not reported here, but the abnormalities observed were consistent with previous reports [17, 18]. Liver enzyme levels were checked in approximately 60% and were moderately elevated at shock, with aspartate aminotransferase levels consistently higher than alanine aminotransferase levels.

All patients would have fulfilled the 2009 WHO criteria for severe dengue, whereas only 939 of 1642 (57%) of the children with sufficient data to allow classification at enrollment would have been categorized as having dengue hemorrhagic fever (DHF). Using all available information from the acute illness and any follow-up visits, 1202 of 1705 (70%) of the patients eventually fulfilled the 4 criteria for DHF, with the remainder classified as having dengue fever by default.

### Outcome

During the 10-year study, only 8 patients died (1 infant and 7 children; Table 3), although 2 additional DSS-associated deaths outside the study were identified from hospital records. In 3 of 8 cases, shock occurred early, on illness day 4. All 8 patients developed profound shock within the first 12 hours, requiring multiple colloid infusions plus inotropic support and with rapid development of significant fluid overload. The interval from admission to death was generally short (median, 34 hours [range, 11–87 hours]), although 1 child with multiorgan failure was taken home moribund after 4 days. Major bleeding requiring transfusion was apparent in 7 of 8 cases before death.

**Table 3.** Selected Clinical and Laboratory Characteristics for the 8 Children Who Died

Characteristic	Patient 1	Patient 2	Patient 3	Patient 4	Patient 5	Patient 6	Patient 7	Patient 8
Demographic characteristics								
Age	11 mo	7 y	6 y	7 y	7 y	13 y	6 y	3 y
Sex	Male	Male	Female	Female	Male	Female	Male	Male
Year of enrollment	2000	2006	2007	2007	2007	2009	2009	2009
At presentation with shock								
Day of illness	5	4	5	5	5	6	4	4
Temperature, °C	39	37	38	39.2	37.5	37	37	37
Pulse rate per min	120	Rapid, weak	120	140	120	152	Rapid, weak	Rapid, weak
Systolic blood pressure, mm Hg	90	95	85	90	85	95	0	90
Pulse pressure, mm Hg	10	15	25	20	10	15	0	20
Bleeding	Petechiae	Petechiae	Petechiae, GI	−	−	Petechiae	Petechiae	−
Abdominal tenderness	+	+	+	+	+	+	−	−
Liver size, cm	4	2	3	2	2	3	1	−
Hematocrit, %	41	NA	NA	42	51	56	53	53
Platelet count, cells/µL	108 000	7000	35 000	38 400	NA	39 000	55 800	97 000
During hospitalization								
Maximum hematocrit, %	41	53	53	46	51	56	53	53
Minimum platelet count, cells/µL	108 000	7000	35 000	13 300	30 400	20 000	7000	17 700
New bleeding	GI	−	−	GI	GI	GI	Epistaxis	GI, epistaxis
Transfusion	+	−	+	+	+	+	+	+
Inotropic support	+	+	+	+	+	+	+	+
Number of colloid boluses	2^a^	2	2	3	5	3	5	5
Total colloid volume, mL/kg	35.8	23.5	11.0	33.1	85.2	54.1	107.5	90.2
Clinical fluid overload	+	+	+	+	+	+	+	+
Hours from admission to death	11	23	24	39	34	37	87	NA^b^
Dengue diagnostic tests								
Serotype	DENV-3	DENV-1	DENV-1	DENV-3	DENV-3	DENV-1	DENV-1	DENV-1
Immune status	Possible primary	Secondary	Secondary	Secondary	Secondary	Secondary	Secondary	Secondary

Abbreviations: DENV, dengue virus; GI, gastrointestinal bleeding; +, yes; −, no; NA, not available.

^a^ Case 1 was enrolled in the fluid randomized controlled trial and received the first bolus of colloid according to the trial randomization.

^b^ Case 8 was taken home 4 days after admission and is presumed to have died. The child was profoundly hypotensive with multiorgan failure at the time of discharge.

Overt organ dysfunction was very uncommon. Other than in association with prolonged shock (Table 3, cases 7 and 8) no patient in the cohort had clinically significant hepatic, renal, or neurological compromise, except for the child with spinal cord hemorrhage and 1 other child with profound shock, liver failure, and coma. These 2 children both eventually made a full recovery with supportive care.

### Dengue Serotypes and Immune Status

**Figure 2.**
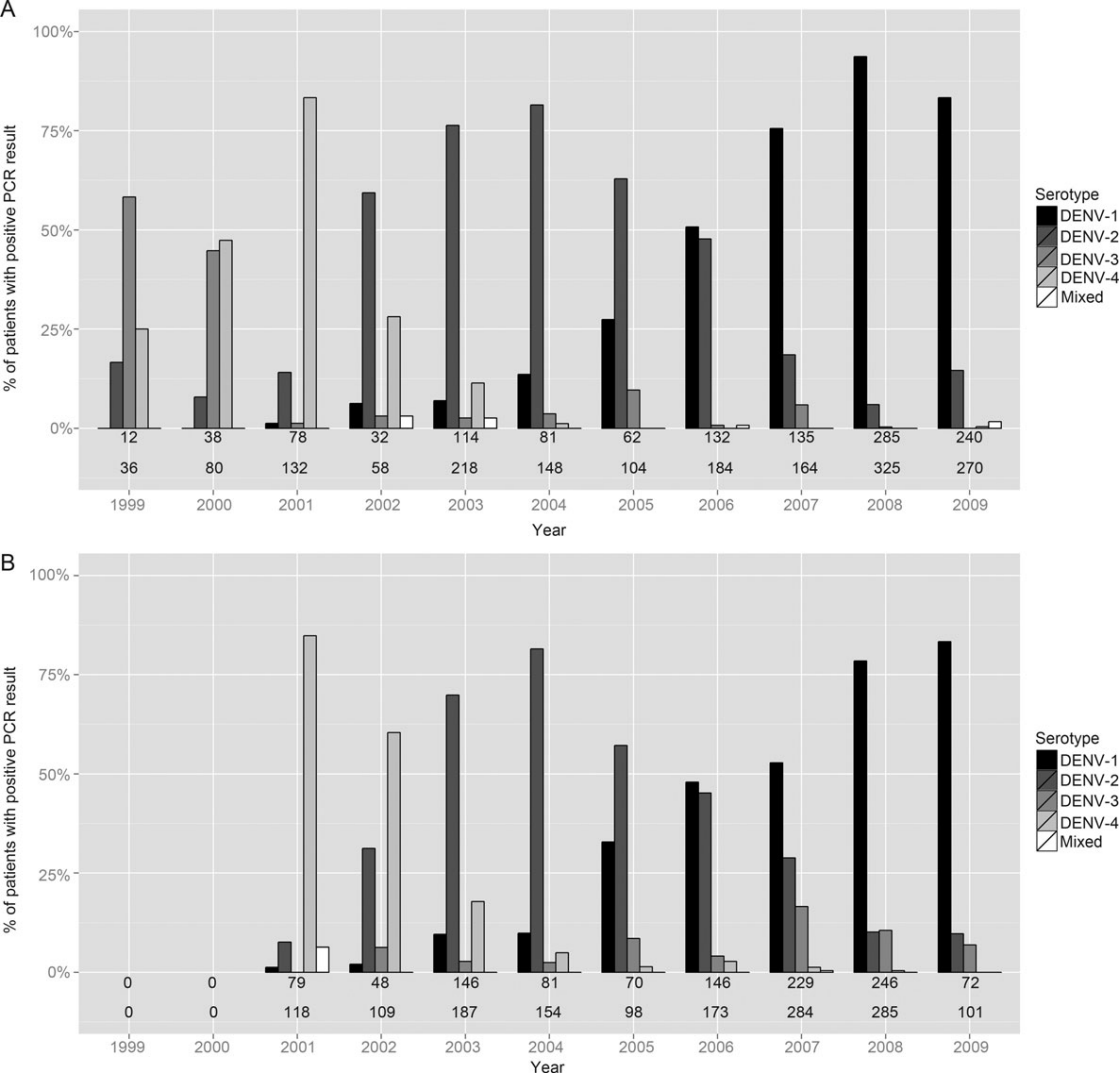
Serotype distributions over time for patients with dengue shock syndrome (*A*), and for children with secondary dengue who were hospitalized at the same facility but did not experience severe complications (*B*). The numbers below each bar are the total number of patients in whom a serotype was identified (upper line), and the total number of patients enrolled into the corresponding study (lower line). Abbreviations: DENV, dengue virus; PCR, polymerase chain reaction.

The relative abundance of dengue serotypes identified in the patient cohort over time is presented in Figure 2*A*. With increasingly sensitive diagnostics, successful serotype identification increased, from 30%–50% initially to >80% after 2007. In 1999, DENV-3 was the most common serotype seen, replaced by DENV-4 peaking in 2001, DENV-2 peaking in 2004, and finally by DENV-1 extending from 2005 to 2009. Almost all patients had an IgG response consistent with secondary infection, although 106 of 1719 (6%) of cases were unclassifiable. The pattern of serotypes observed was very similar to that seen among 1509 children with secondary dengue without shock enrolled into a separate observational study between 2001 and 2009 (Figure 2*B*; unpublished data).

Only 6 cases had clear primary infections, including 4 infants aged <18 months, and 2 children aged 7 and 12 years (Table 4). Immune status was suggestive of primary disease in 4 of the 5 other children aged <18 months (no information in 1 case), whereas all 157 children aged 18–60 months with classifiable immune status had secondary dengue. Infants may be underrepresented in the cohort, however, as many parents elect to take young children to 1 of 2 specialist pediatric hospitals nearby. All of the patients with definite primary cases recovered, although 2 infants required colloid infusions. However, one 11-month-old boy with indeterminate/possible primary dengue died with profound shock.

**Table 4.** Selected Clinical and Laboratory Characteristics for the 6 Primary Dengue Cases

Characteristic	Patient 1	Patient 2	Patient 3	Patient 4	Patient 5	Patient 6
Demographic characteristics						
Age	4 m	11 m	13 m	7 y	11 m	12 y
Sex	Male	Female	Male	Male	Female	Male
Year of enrollment	2008	2008	2008	2008	2009	2009
At presentation with shock						
Day of illness	5	5	5	5	5	6
Temperature, °C	37.5	37	37	37	37	37
Pulse rate per min	148	Rapid, weak	168	140	Rapid, weak	110
Systolic blood pressure, mm Hg	50	70	90	90	80	90
Pulse pressure, mm Hg	20	20	15	20	20	20
Bleeding	Petechiae	Petechiae	Petechiae	−	Petechiae	Petechiae
Abdominal tenderness	+	+	+	+	−	−
Liver size, cm	3	1	4	3	2	1
Hematocrit, %	36	47	50	56	45	46
Platelet count, cells/µL	40 000	19 000	77 000	81 400	37 900	66 000
During hospitalization						
Maximum hematocrit, %	36	47	50	56	45	47
Minimum platelet count, cells/µL	24 000	9000	45 800	61 000	28 400	53 000
New bleeding	−	−	−	−	−	−
Number of colloid boluses	0	2	1	0	0	0
Total colloid volume, mL/kg	0	25.0	25.3	0	0	0
Clinical fluid overload	−	−	+	−	−	−
Outcome	Recovery	Recovery	Recovery	Recovery	Recovery	Recovery
Dengue diagnostic tests						
IgG by day of illness	Day 5: (−)	Day 5: (−)	Day 5: (−)	Day 5: (−)	Day 5: (−)	Day 6: (−)
	Day 8: (−)	Day 8: (−)	Day 8: (−)	Day 8: (−)	Day 8: (−)	Day 9: (−)
Serotype	DENV-2	DENV-1	Negative^a^	DENV-1	DENV-1	DENV-1

Abbreviations: +, yes; −, no; DENV, dengue virus; IgG, immunoglobulin G.

^a^ Diagnosis based on positive dengue immunoglobulin M capture enzyme-linked immunosorbent assay on samples taken on days 5 and 8.

## DISCUSSION

Here we present the first comprehensive description of the clinical features of DSS in children, using data gathered prospectively over 10 years on 1719 patients managed in a single Vietnamese institution. More than 95% of all children admitted with DSS during the study period were evaluated. Because prior shock resuscitation might confound the clinical picture, we focused on direct admissions only. A few cases were missed, including 2 children who died, but overall the results are representative of the clinical spectrum of patients with DSS admitted directly to a busy hospital in a hyperendemic region.

Apart from infants aged <18 months, virtually all children had secondary dengue, in line with established concepts of pathogenesis [19, 20]. We observed DSS caused by all 4 dengue serotypes during the 10-year study; the pattern of serotype replacement over time was similar to that seen among children with secondary infections enrolled in a contemporaneous study of hospitalized dengue without severe manifestations, and also to the relative virus prevalence identified by passive surveillance in southern Vietnam during the same time period [21]. Thus, the viruses associated with DSS appear to be representative of the virus population affecting the wider community, with no evidence that a particular serotype contributes to a greater risk for shock. Notably, however, 3 of 8 deaths were associated with DENV-3, although the total number of DENV-3 infections identified was small. Because a number of interacting host and viral factors influence an individual's propensity to develop severe vascular leakage [19], only very detailed studies can establish whether particular viral characteristics do confer an increased risk for DSS or death.

The clinical signs and symptoms documented were generally consistent with empirical descriptions of DSS [4]. However, 9% of all patients were still febrile at presentation. Increased permeability commences during the febrile phase, but shock develops only when leakage exceeds the capacity of the homeostatic compensatory mechanisms to maintain adequate plasma volume [22, 23], potentially compounded by functional cardiac impairment [24]. Although defervescence and shock are often temporally linked, it is important that clinicians managing suspected dengue cases understand that DSS can occur earlier. Identification of more reliable warning signs of likely deterioration would be useful both for individual case management and to facilitate effective use of limited healthcare resources.

In agreement with other studies [7, 25], a considerable number of DSS patients had no bleeding during the illness. Severe bleeding was uncommon and primarily from the gastrointestinal tract, although massive soft tissue bleeding necessitating transfusion occurred in 4 children. Again consistent with other studies [25, 26], almost one-third of cases did not achieve the WHO 1997 classification for DHF, mainly due to failure to fulfill the hemorrhage and/or plasma leakage criteria as thrombocytopenia was almost universal. If positive, a tourniquet test might have allowed classification as DHF rather than dengue fever in 357 additional cases, but several studies have demonstrated poor utility of the test in clinical practice, and it is infrequently performed in Vietnam [25, 27]. We did not perform radiological investigations to identify plasma leakage unless clinically indicated, reflecting real-world practice. Thus, diagnosis of leakage typically rested upon demonstration of hemoconcentration, yet hemoconcentration below the WHO threshold of 20% has been noted previously in DSS patients [11]. Because patients must be treated according to their actual clinical status, it is apparent that the 1997 WHO classification system is not suitable for individual case management in real time.

The case fatality rate was extremely low (0.5%). Most patients recovered well with the standard crystalloid regimen or following a single colloid bolus, and requirement for additional colloid therapy, inotropic support, and/or blood products was infrequent. Prompt diagnosis and immediate admission to PICU with management coordinated by a highly experienced team undoubtedly contributed to this favorable outcome. In line with WHO principles [4], the unit operates a generally conservative fluid management policy after initial resuscitation, relying on frequent clinical assessments and regular ward-based hematocrit measurements to limit fluid administration to the minimum required, reducing the risk of fluid overload. However, our study focused on direct admissions, and it is clear that external referrals with prolonged shock or established fluid overload are more difficult to manage and have correspondingly higher mortality rates [8]. Only a very small number of patients with confirmed primary dengue were included in the cohort, and all recovered quickly without notable complications. However, 1 death occurred in a suspected primary case, underlining the view that primary dengue can result in severe and even fatal disease [28–30]. Given that immune status could not be defined in 6% of patients, some primary cases might have been missed, but the number is likely to be small.

In summary, we present a comprehensive clinical description of DSS in a large cohort of Vietnamese children. We demonstrate that with prompt intervention and assiduous clinical care by experienced staff, the outcome of this potentially fatal condition can be excellent. As the emerging dengue pandemic spreads to new geographical locations, it is important that this accumulated experience be translated into practical advice for clinicians newly exposed to this severe complication of a common disorder.

## Supplementary Data

Supplementary materials are available at *Clinical Infectious Diseases* online (http://cid.oxfordjournals.org). Supplementary materials consist of data provided by the author that are published to benefit the reader. The posted materials are not copyedited. The contents of all supplementary data are the sole responsibility of the authors. Questions or messages regarding errors should be addressed to the author.

Supplementary Data
