# Supplementary material for: Clinical Characteristics of Dengue Shock Syndrome in Vietnamese Children: A 10-Year Prospective Study in a Single Hospital
Source: Clin Infect Dis. 2013 Sep 17;57(11):1577–86. doi: 10.1093/cid/cit594 (PMC3814826; doi:10.1093/cid/cit594)
Supplement: Supplementary Data [file supp_57_11_1577__index.html]

Clinical characteristics of dengue shock syndrome in Vietnamese children; a 10-year prospective study in a single hospital — Clinical Characteristics of Dengue Shock Syndrome in Vietnamese Children: A 10-Year Prospective Study in a Single Hospital — Supplementary Data 

# Clinical Characteristics of Dengue Shock Syndrome in Vietnamese Children: A 10-Year Prospective Study in a Single Hospital

## Supplementary Data

Supplementary Data

**Files in this Data Supplement:**

- Supplementary Data - Doc file
