# Supplementary material for: Clinical Characteristics of Dengue Shock Syndrome in Vietnamese Children: A 10-Year Prospective Study in a Single Hospital
Source: Clin Infect Dis. 2013 Sep 17;57(11):1577–86. doi: 10.1093/cid/cit594 (PMC3814826; doi:10.1093/cid/cit594)
Supplement: Supplementary Data [file supp_cit594_cit594supp.doc]

**Appendix 1**

1. **Case definitions for dengue haemorrhagic fever (DHF) and dengue shock syndrome (DSS) as per the WHO 1997 guidelines** [1]

**Case definition for DHF-** ALL four of the following criteria must be fulfilled:

1. Fever, or history of acute fever, lasting 2-7 days, occasionally biphasic.
2. Evidence of haemorrhagic tendency - at least ONE of the following:
   - A positive tourniquet test
   - Petechiae, ecchymoses or purpura
   - Bleeding from the mucosa, gastrointestinal tract, injection sites or other locations
   - Haematemesis or melaena.
3. Thrombocytopenia (<= 100,000 cells per mm^3^)
4. Evidence of plasma leakage - at least ONE of the following:
   - A rise in the haematocrit >= 20% above the mean for age, sex and population
   - A drop in the haematocrit following volume replacement >=20% of baseline
   - Signs of plasma leakage such as pleural effusion, ascites and hypo-proteinaemia.

**Case definition for DSS -** ALL 4 criteria for DHF must be present plus evidence of circulatory failure manifested by:

- Rapid and weak pulse, and
- Narrow pulse pressure (< 20 mmHg)

or manifested by:

- Hypotension for age, and
- Cold, clammy skin and restlessness.

Hypotension for age is defined as a systolic blood pressure < 80 mmHg for those below 5 years of age, or < 90 mmHg for those greater than or equal to 5 years of age.

1. **Criteria for dengue and severe dengue as per the 2009 WHO guidelines** [2]

**Criteria for dengue with or without warning signs:**

1. Probable dengue - live in/travel to a dengue endemic area, with fever and at least 2 of the following criteria:
   - Nausea, vomiting
   - Rash
   - Aches and pains
   - Tourniquet test positive
   - Leucopenia
   - Any warning sign
2. Laboratory confirmation is encouraged when there are no signs of plasma leakage

**Warning signs:**

- - Abdominal pain or tenderness
  - Persistent vomiting
  - Clinical fluid accumulation
  - Mucosal bleed
  - Lethargy, restlessness
  - Liver enlargement > 2 cm
  - Increase in haematocrit level concurrent with rapid decrease in platelet count

**Criteria for severe dengue:**

1. Severe plasma leakage leading to
   - Shock (DSS)
   - Fluid accumulation with respiratory distress
2. Severe bleeding - as evaluated by clinician
3. Severe organ involvement
   - Liver: AST or ALT >= 1000 IU/L
   - Nervous system: impaired consciousness
   - Heart or other organ dysfunction

**Appendix 2**

**Dengue diagnostics**

During the study period the diagnostic laboratory used a number of different serological tests, following the manufacturer’s instructions for commercial kits (Dengue Duo IgM and IgG Capture ELISA, PanBio, Australia), or established standard operating procedures for in-house methods.[3] A laboratory-confirmed case was defined by seroconversion on the capture ELISA or by detection of DENV RNA in plasma.[4,5] Patients in whom the dengue-specific IgM was already high at onset of shock were also considered as confirmed dengue provided the overall clinical picture was consistent with DSS and no alternative diagnosis was established. Cases were defined as dengue-negative if the enrolment RT-PCR and paired serology specimens were all negative.

Serological definitions for primary versus secondary infections commonly rely on the ratio of IgM/IgG, but may give varying results depending on the test used and the day of illness when the specimen is obtained. Given that a number of different sero-diagnostic tests were employed during the study-period we used the following simple definitions: a positive dengue-specific IgG on or before day 7 of illness defined a secondary infection; two negative dengue-specific IgG results defined a primary infection provided the second sample was obtained during the second week of illness; all other patients, mainly with single specimens or late convalescent specimens, were considered unclassifiable.

Cross reactivity with flaviviruses that co-circulate in the region may influence the results of IgG serology in particular – in Vietnam Japanese encephalitis (JE) virus is known to circulate and to cause sporadic cases of meningo-encephalitis. However, although inactivated JE vaccine was introduced in 1997 for use in high-risk areas, southern Vietnam is not considered to be high-risk and JE vaccination is not part of the local Expanded Program of Immunization. Vaccination became available privately in Ho Chi Minh City from around 2005-2006 onwards but uptake remains sporadic. Although we did not include specific serological tests for JE virus to assess cross-reactivity, due to the low vaccine coverage locally it is unlikely that JE vaccination affected the identification of secondary dengue cases in this study. Symptomatic disease caused by JE virus is primarily neurological and very unlikely to mimic DSS. None of the patients in the cohort had a past history of serious neurological disease, but it is possible that recent asymptomatic or pauci-symptomatic JE virus infection influenced the serological responses we documented but the number of cases affected is likely to be small.

**References:**

1. World Health Organization. Dengue haemorrhagic fever: diagnosis, treatment, prevention and control. Geneva: World Health Organization, **1997**.

2. World Health Organization. Dengue Guidelines for Diagnosis,Treatment, Prevention and Control. France: World Health Organization, **2009**.

3. Cardosa MJ, Wang SM, Sum MSH, Tio PH. Antibodies against prM protein distinguish between previous infection with dengue and Japanese encephalitis viruses. BMC Microbiol, **2002**; 2:9.

4. Lanciotti RS, Calisher CH, Gubler DJ, Chang GJ, Vorndam a V. Rapid detection and typing of dengue viruses from clinical samples by using reverse transcriptase-polymerase chain reaction. J Clin Microbiol, **1992**; 30:545–51.

5. Shu P, Chang S, Kuo Y, et al. Development of group- and serotype-specific one-step SYBR green I-based real-time reverse transcription-PCR assay for dengue virus. J Clin Microbiol, **2003**; 41:2408–16.
